# Effects of ketamine on rat social behavior as analyzed by DeepLabCut and SimBA deep learning algorithms

**DOI:** 10.3389/fphar.2023.1329424

**Published:** 2024-01-10

**Authors:** Piotr Popik, Ewelina Cyrano, Diana Piotrowska, Malgorzata Holuj, Joanna Golebiowska, Natalia Malikowska-Racia, Agnieszka Potasiewicz, Agnieszka Nikiforuk

**Affiliations:** Behavioral Neuroscience and Drug Development, Maj Institute of Pharmacology, Polish Academy of Sciences, Kraków, Poland

**Keywords:** NMDA receptor antagonist, DeepLabCut, SimBA, EQIPD quality system, ethology

## Abstract

Traditional methods of rat social behavior assessment are extremely time-consuming and susceptible to the subjective biases. In contrast, novel digital techniques allow for rapid and objective measurements. This study sought to assess the feasibility of implementing a digital workflow to compare the effects of (*R,S*)-ketamine and a veterinary ketamine preparation Vetoquinol (both at 20 mg/kg) on the social behaviors of rat pairs. Historical and novel videos were used to train the DeepLabCut neural network. The numerical data generated by DeepLabCut from 14 video samples, representing various body parts in time and space were subjected to the Simple Behavioral Analysis (SimBA) toolkit, to build classifiers for 12 distinct social and non-social behaviors. To validate the workflow, previously annotated by the trained observer historical videos were analyzed with SimBA classifiers, and regression analysis of the total time of social interactions yielded *R* ^2^ = 0.75, slope 1.04; *p* < 0.001 (N = 101). Remarkable similarities between human and computer annotations allowed for using the digital workflow to analyze 24 novel videos of rats treated with vehicle and ketamine preparations. Digital workflow revealed similarities in the reduction of social behavior by both compounds, and no substantial differences between them. However, the digital workflow also demonstrated ketamine-induced increases in self-grooming, increased transitions from social contacts to self-grooming, and no effects on adjacent lying time. This study confirms and extends the utility of deep learning in analyzing rat social behavior and highlights its efficiency and objectivity. It provides a faster and objective alternative to human workflow.

## 1 Introduction

Rat’s social life (Lore and Flannelly, 1977) is extremely complex (Barnett, 1958; Calhoun, 1962; Whishaw and Kolb, 2005) and sensitive to pharmacological manipulations; e.g., (Panksepp et al., 1979; Popik et al., 1992).

Historically, rodent social behaviors have been classified by the trained observers who scored behaviors either at the time the test was being conducted, or off-line, by watching the beta-cam/VHS tapes or computerized videos. In a typical scenario, the trained observer scored social behavior using the pencil, paper and a stopwatch, free software like BORIS (https://www.boris.unito.it/); see (Friard et al., 2016), Behaview (http://www.pmbogusz.net/?a=behaview), JWatcher (https://www.jwatcher.ucla.edu/), commercial programs like Observer^®^ (Noldus, Wageningen, Netherlands) or other software. Such approach, however, is extremely time-consuming, as it takes human annotators 3–4 × the video’s duration to annotate; for long recordings, there is also risk of drops in annotation quality due to drifting annotator attention (Segalin et al., 2021). In addition, it is also prone to subjective bias and require deep experience and knowledge of social behavior (Niesink and Van Ree, 1982; Datta et al., 2019; Buhler et al., 2023), and requires marking or painting the body parts of the interacting animals.

While there have been attempts to automate social behavior scoring, e.g., using EthoVision^®^ [Noldus, Wageningen, Netherlands (Sams-Dodd, 1995)], recent advances in computer vision and deep learning based toolkits as well as the economical availability of the fast computer graphic cards, enabled to analyze videos of the rodent behavior almost semi-automatically. Specifically, behavioral analyses offered by the open source DeepLabCut (https://github.com/DeepLabCut) marker-less pose-estimation toolkit (Mathis et al., 2018; Nath et al., 2019; Lauer et al., 2022) have vastly facilitated the analytical workflow. This is because DeepLabCut offers the generation of neural networks (models) representing interacting animals’ body parts in time and space. This freely available and easy to use–even by a computer non-expert researcher–software, allows for semi-automatic tracking of animals’ movements.

A necessary second step in behavioral analysis of social encounters requires a post-processing software that, using numerical data representing animals’ body parts in time and space (i.e., the CSV files provided by the DeepLabCut) would classify social behavior. In this context, Simple Behavioral Analysis (SimBA https://github.com/sgoldenlab/simba) free Python’s toolkit, of a user-friendly interface, and not requiring programming knowledge, constitutes another analytical break-through (Nilsson et al., 2020). Of note, both toolkits are supported by the community of experts and users, and are continuously developed and enhanced in their features. Their use is supposed to reduce the analysis time and to avoid human observer’s bias.

Rats’ social behavior could be studied for a variety of reasons. These include modeling certain symptoms of psychoses. In this respect, the antagonists of glutamate NMDA receptor are thought to (re)produce certain symptoms of schizophrenia most closely; for a review see (Bubenikova-Valesova et al., 2008). NMDAR antagonist-based psychosis model is not only easy to use, produce the most reliable results, but also models the “negative” symptoms, like social withdrawal, with the highest face, and predictive validity. For instance, it has been shown that NMDAR antagonist phencyclidine (Steinpreis et al., 1994; Sams-Dodd, 1995; Koros et al., 2007) as well as (+)-MK-801, memantine and the metabotropic glutamate receptor subtype 5 (mGluR5) antagonist, MTEP (Koros et al., 2007) reduce social behavior of rats.

Similar effects have been shown for another dissociative anesthetic and NMDAR antagonist, ketamine (Becker et al., 2003; Becker and Grecksch, 2004; Koros et al., 2007). Indeed, ketamine appears as a golden standard in producing social withdrawal in rats (Silvestre et al., 1997; Koros et al., 2007).

This laboratory has substantial data record in using ketamine to induce deficits in rat social behavior and in investigating ways to reduce them. For instance, in 2013 we reported that acute administration of atypical antipsychotic amisulpride (3 mg/kg) ameliorated ketamine (20 mg/kg, IP given 30 min before testing) - induced disruption of social behavior (Nikiforuk et al., 2013). In the following work (Holuj et al., 2015) we confirmed these effects and additionally showed an inefficacy of dopamine D2 receptor antagonists sulpiride (20 or 30 mg/kg) and haloperidol (0.2 mg/kg) in reversing ketamine-induced social withdrawal. As in the earlier work, ketamine (20 mg/kg, IP) was given 30 min before testing. Another investigation from this laboratory demonstrated dose-related effects of ketamine (20 but not 10 mg/kg significantly reducing social behavior) and of another veterinary dissociative anesthetic and NMDAR antagonist, tiletamine (Popik et al., 2017). We also investigated the effects of two novel potential antipsychotic compounds and of clinically used aripiprazole, risperidone, clozapine, haloperidol, and ziprasidone on ketamine-induced social withdrawal (Zajdel et al., 2018).

In all these experiments we used the veterinary preparation of ketamine hydrochloride racemate (Vetoquinol). This medication contains also a minute (3 mg/mL) amount of the sedative, weak local anesthetic, antibacterial and antifungal compound chlorobutanol hemihydrate. For surgery, the preparation is typically used at the anesthetic doses (≥150 mg/kg). Thus, when the Vetoquinol ketamine is used at ∼7 times lower doses (20 mg/kg) for other purposes, including social behavior experiments, the amount of chlorobutanol hemihydrate is unlikely to produce sedation. However, we decided to examine this hypothesis experimentally, by directly comparing the effects of Vetoquinol ketamine with the effects of pure (*R,S*)-ketamine.

The first aim of this work focused on finding out how the top-modern computerized analyses match the human scores of the historical, i.e., already analyzed social behavior experiments from this laboratory. This was possible, due to the availability of historical videos and the raw data with human annotations, stored on the computer disks.

As the initial analyses revealed remarkably similar patterns of social behavior and confirmed ketamine-induced deficits, we compared recently recorded social behavior of rats treated with veterinary ketamine preparation Vetoquinol, with the pure (*R,S*)-ketamine. This was done to examine whether our published data could have been confounded by the impurity of ketamine. For this novel analysis, only digital workflow was used.

## 2 Methods

### 2.1 Digital workflow

We provide a detailed workflow allowing easy reproduction of the steps leading to the semi-automated analysis of behavioral data. While both the DeepLabCut (https://github.com/DeepLabCut) and SimBA (https://github.com/sgoldenlab/simba) websites offer comprehensive details of the installation and use of the respective toolkits, we describe the methods in a way allowing less computer-oriented researchers an easy step-by-step guide do analyze novel data.

#### 2.1.1 Training DeepLabCut network of two interacting rats

**FIGURE 1:**
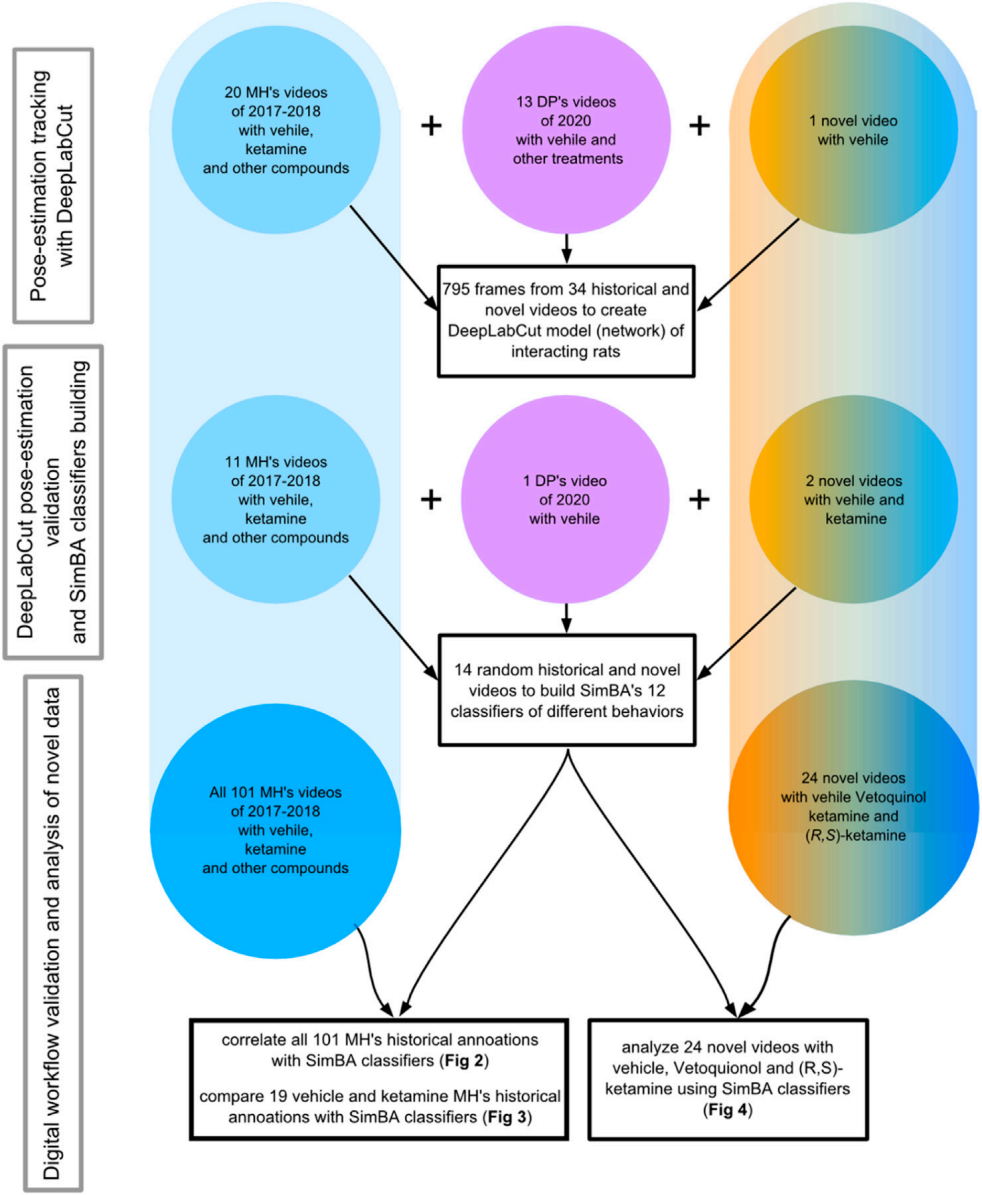
Presented are the dates, sources and numbers of videos used for DeepLabCut network training (upper), for DeepLabCut pose-estimation’s validation and SimBA’s classifiers’ building (middle), as well as for the statistical analyses of the historic videos (bottom left) and for the novel videos’ analysis (bottom right). Blue color indicates Vetoquinol ketamine data inclusion while the orange color shows videos of rats treated with (*R, S*)-ketamine. The pink color shows videos of rats not treated with ketamine. MH and DP are the initials of the highly trained observers.

Figure 1 shows the choice of a set of random 34 historical and recent videos of two interacting male or female rats, recorded in our laboratory between years 2017 and 2023. The videos were of rats weighing 250–350 g, subjected to different treatments, recorded by different researchers in mostly similar but not identical experimental conditions. This was done to create digital workflow as universal for our experimental conditions, as possible (Hardin and Schlupp, 2022). What was common was that every scene contained only two interacting white rats (Sprague-Dawley or Wistar strain) placed in the black “open field” arena for 10 min. The sizes of the arena slightly varied across experiments and were 55 × 65, 57 × 67 or 65 × 65 cm (W x L); the height was always 30 cm. These sizes corresponded to the size of the arena used in the present experiment (length x width x height: 57 × 67 × 30 cm).

While historical videos were recorded at 15–25 frames per second (fps) using analog CCTV cameras and commercial Noldus MPEG recorder or AnyMaze^®^ programs, their frame rate was set with freely available ffmpeg program (https://ffmpeg.org/) to the common 12 fps, as this was the minimum effective recording rate, considering duplicated frames in the proprietary video format files provided by AnyMaze^®^ program.

To quote Hardin and Schlupp, (2022) when selecting a subset of videos to extract frames from, and extracting the frames themselves for marking, it is important to aim for as much variety in behavior, posture, individuals used, backgrounds, etc. as is possible within the dataset. Thus, a set of 795 random frames (PNG images) taken from various 34 videos was typically cropped to 546 × 512 pixels with ffmpeg program and used to train the DeepLabCut network. To create the training set, every training frame was manually labeled with a total of 18 points: for every animal, the nose, left eye, right eye, head, left shoulder, right shoulder, back, pelvis and anogenital region was marked on the computer screen. This was done over several days using DeepLabCut “napari” plugin, on a desktop PC with Windows 11 equipped with the Nvidia RTX 4090 graphic card and the GUI version 2.3.5 of DeepLabCut running on Python 3.8.16.

To achieve the best possible model, we ran several DeepLabCut “shuffles”, gradually increasing the number of annotated frames, eliminating badly recognized frames, and varying the number of iterations. Other DeepLabCut’s variables were set at default values. This was done iteratively over several weeks on the Nvidia DGX A100 station running Ubuntu 22 Linux, Python 3.9.15 (main, 4 Nov 2022, 16:13:54), IPython 8.6.0, and DeepLabCut version 2.3rc2.

Considering the DeepLabCut’s accuracy of body parts recognition (or human inaccuracy in marking them: note that all body parts of both interacting rats have to be ideally “seen” in each frame to achieve reasonable predictions of their locations in time and space), we corrected or deleted inaccurately recognized frames, so that a total of 745 frames was used to train the multi-animal model based on the ResNet_v1_50 neural network.

While we used a multi-animal approach, we did not differentiate animal A from animal B. As pointed by Hardin and Schlupp, (2022), DeepLabCut performs tracking, which could be further improved by manual refinement. The program struggles the most when two similar individuals overlap in the same frame, leaving the software guessing “who is who” when the overlapping ends. The user may correct the overlapping frames by refining portions of videos and retraining the model. However, we found the process of manual refinement extremely time-consuming. Bearing in mind that in this work DeepLabCut analyzed 115 historical (human-annotated) and 24 novel videos, each of 10-min length, that is ∼ 23 h of recordings, we considered the output of clearly distinguished 2 animals, whose “identities” were sometimes interchanged, as satisfactory enough for further analyses. While the lack of animal identity produced some problems with the interpretation of the behavioral transitions (see below), present workflow did not require to know “who is who” because both animals were always given the same treatment, were of the same sex and their weight was matched.

Following 200,000 iterations, and several hours of computer engagement, the final “shuffle” was characterized with the train and test errors of 3.01 and 4.33 pixels, respectively, and 2.9 and 4.32 pixels respectively with P cutoff of 0.6. The average Euclidean distance to GT per body part (test-only) were: anogenital 3.52, back 4.80, head 4.73, left eye 3.49, left shoulder 5.09, nose 3.11, pelvis 5.15, right eye 3.59, and right shoulder 5.19 pixels.

#### 2.1.2 Using DeepLabCut model to obtain numerical data of interacting rats

In the next step, 14 randomly chosen videos, (other than the 34 videos used for DeepLabCut network training), recorded over years 2017–2023 were analyzed with the network created by DeepLabCut to further validate the model. When playing videos on a computer screen at reduced speed with the free mpv program (https://mpv.io/), we noted that the body parts were marked by DeepLabCut in the way the observer would mark them, and that the incidents of interchanging a body part between rats, or of “loosing” the animal from the view, were sporadic. Most of these videos were of the full 10-min length but five contained only shorter (2-min–4-min) fragments rich in behaviors like fighting or adjacent lying, as these were not seen on all recordings. Ffmpeg program was used to dissect interesting parts of the videos.

As a result, DeepLabCut analysis provided numerical data (CSV files) representing every body part position in space and time; these 14 files were used by SimBA toolkit in the next steps.

#### 2.1.3 Using DeepLabCut numerical data to create SimBA classifiers of different behaviors

Following DeepLabCut analysis, the 14 training videos and their corresponding DeepLabCut filtered CSV files were loaded to the Simple Behavioral Analysis (SimBA) toolkit version 1.72.2 on the Windows 11 PC running Python 3.6.13. Except undersampling (see below), all other parameters were set to default values. All these 14 videos were manually annotated within SimBA, as showing or not showing one of 12 behaviors: 1 **adjacent lying** [the time of side by side contact or “huddling”; see (Morley et al., 2005; Ando et al., 2006)], 2 **anogenital sniffing** (one rat sniffing the anogenital region of the conspecific), 3 **crawling** (one rat moving over or under the conspecific), 4 **fighting** [mostly aggressive grooming: one rat chasing another then, while pinning down a conspecific or holding it with the forepaws: licking, chewing the fur of the conspecific, or punching; see (Barnett, 2001; Goodwin et al., 2020; Nilsson et al., 2020; Schweinfurth, 2020)], 5 **following** (active movement of two individuals; one chasing and approaching another), 6 **grooming** [known also as allogrooming: cleaning the fur or amicably scratching, licking and chewing the fur of the conspecific, motionless (Schweinfurth, 2020)], 7 **mounting** (climbing or standing on the back of the conspecific), 8 **nosing** [rats touching each other with their noses, while stretching their body slightly (Sams-Dodd, 1995)], 9 **rearing** (one or both animals standing on their hind legs), 10 **self-grooming** [cleaning the fur or scratching: rapid movements of the head towards the own body (Sams-Dodd, 1995)] and 11 **sniffing** (sniffing or touching the body of the conspecific). We also trained SimBA to recognize (12): **immobility** (a drug side-effect: one or two rats resting motionless, not in a social contact). The last parameter was not considered as representing stereotypy: it was rather a sign of the “hanging” or “suspending”, typically observed in rats treated with NMDAR antagonists (Koros et al., 2007).

The choice of these behaviors was based on the ethological observations (Barnett, 1958; Barnett, 2001; Calhoun, 1962; Sams-Dodd, 1995; Whishaw and Kolb, 2005; Goodwin et al., 2020; Nilsson et al., 2020; Schweinfurth, 2020) and represents highly characteristic set of behaviors of the same-sex *laboratory* rats in dyadic encounters. It should be emphasized that the “true” fighting (two rats tumble, roll over the ground while holding, kicking, boxing and punching each other) is mostly observed among *wild* rats; see (Schweinfurth, 2020) and references above. Adjacent lying (motionless side by side contact or “huddling”) is regarded as a characteristic feature of the psychoactive MDMA “ecstasy” compound’s action (Morley et al., 2005; Ando et al., 2006).

Representative examples of the 12 scored behavioral categories are shown at Figure 3, bottom.

The SimBA toolkit allowed also for the assessment of two additional measures that were not analyzed previously in our earlier work, including motor activity (movement in cm, shown on Figures 3I, J; Figures 4F, G). In addition, SimBA allowed for Forward Spike Time Tiling Coefficient (FSTTC) analysis (Lee et al., 2019), which detected the sequences of dyadic pairwise behaviors being present within a given time window, i.e., the proportion of behavior B onsets that fall inside delta-t following the onset of behavior A. Because with the present setup, DeepLabCut and SimBA did not differentiate the individual animals, the transitions so calculated tell only that the behavior A was followed by the behavior B, regardless of which of the animals has initiated a given behavior. This could be viewed as a limitation of present approach. For the analyses of temporary data, we set delta-t = 2,000 ms (Lee et al., 2019). For the sake of clarity, the graphs on Figures 4M–O represent coefficients > 0.5 (i.e., show only the most frequent transitions of all behaviors) while Figure 4P shows all the social contact behaviors leading to self-grooming only, with coefficients > 0 (i.e., show all of behaviors leading to self-grooming in all investigated 3 treatment groups altogether).

In the set of 14 SimBA training videos, over several weeks, we inspected all the 71,984 frames, detected 29,833 frames containing some behavior, and identified, depending on a given behavior, as little as 288 (mounting) and as much as 12,265 (rearing) frames containing behaviors of interest. Thus, instead of importing human annotations of historic videos from the other (Noldus Observer^®^) software, which could result in building identical classifiers for the digital workflow, every frame was *de novo* annotated within SimBA toolkit. These annotated training sets of CSV files served SimBA to create behavioral models (classifiers) for further automatic detection.

Every classifier has been iteratively assigned with its **detection threshold** probability derived from the prediction curves, (how sure the computer must be, to classify a given frame as containing a given behavior). These values were then tweaked by inspecting resulting videos to provide even better detections. In addition, every classifier has been assigned with its **minimal bout length** so that, some classifiers, like *nosing* was required to be present for 50 ms, while *adjacent lying* was required to be present for 750 ms to be “caught” by a classifier. The classifiers were created using the random forest approach, entropy criterion, 20% of the training data, and various (0–32) random undersample parameters, which were gradually titrated. Specifically, as the frequencies of different behaviors varied (see above), for the less frequent behaviors, the higher undersample values provided the classifiers with higher F1 values, thus better modelling a given behavior presence (Nilsson et al., 2020). In short, SimBA’s classifiers with the highest F1 values were used as the models used to detect behaviors on the novel videos.

#### 2.1.4 Validation: comparing human annotations with SimBA classifiers in detecting rats social behaviors on historical videos

**FIGURE 2:**
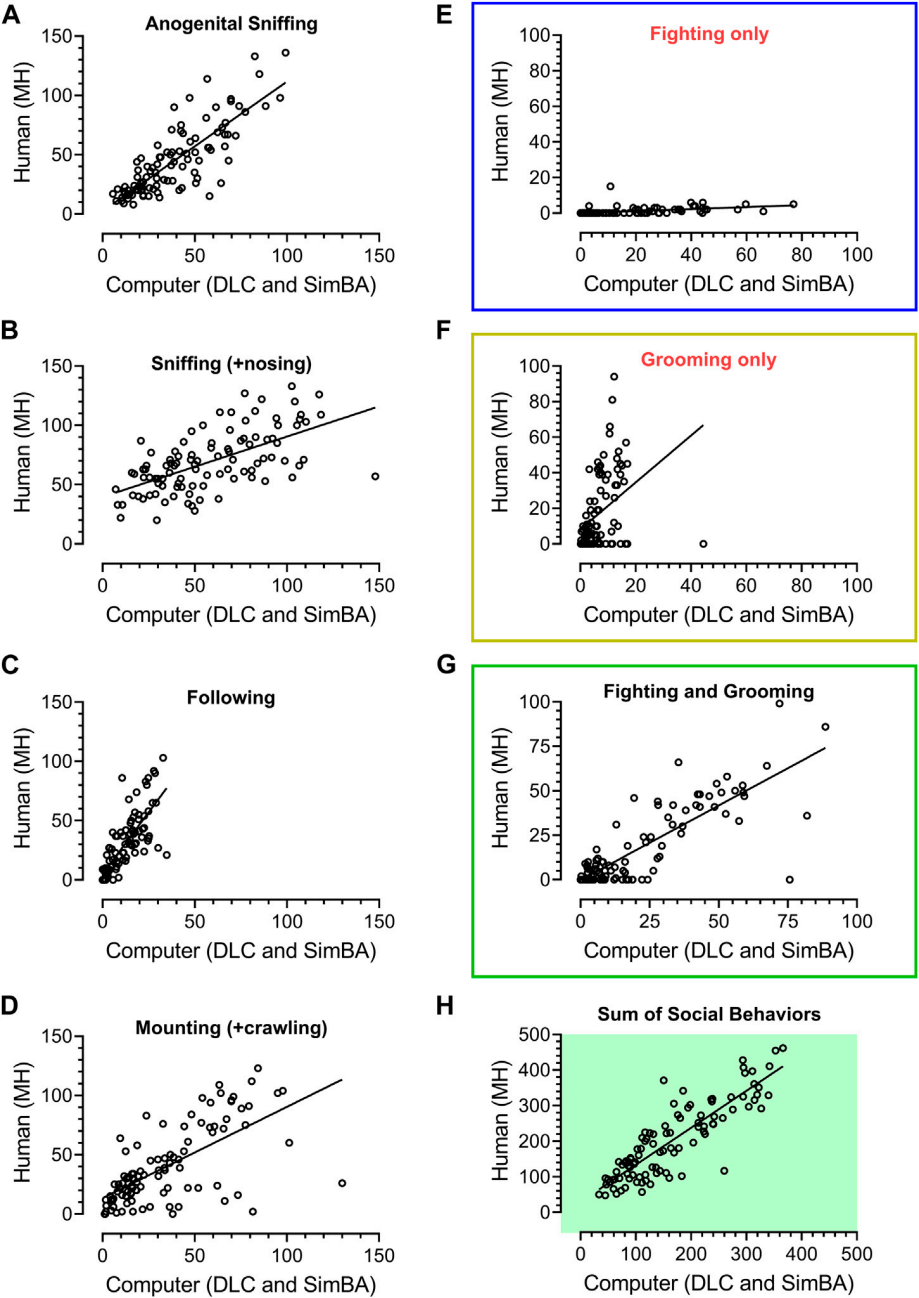
Regression analyses of 101 unpublished historical videos with rat social behavior analyzed in our laboratory over the years 2017–2018 by the experienced researcher (MH) and analyzed by a digital workflow (DeepLabCut and SimBA toolkits). The axes represent time of social behavior in seconds. The animals were treated with vehicle, Vetoquinol ketamine and other compounds. All specific behavioral categories **(A–G)** as well as their sum **(H)**, yielded significant *R* ^2^ values (see Results). While fighting’s **(E)** and grooming’s **(F)** regression coefficients were significant (*p* < 0.001), it was apparent that the human has classified fighting-like behavior less frequently than SimBA, and grooming-like behavior more frequently than SimBA. Bearing similarities between these two behaviors (mostly conspecific’s fur chewing), likely difficult to distinguish for the human and SimBA, we decided to combine them into a fighting + grooming category **(G)**, which yielded *R* ^2^ = 0.67, slope of 0.83 and *p* < 0.001.

**FIGURE 3:**
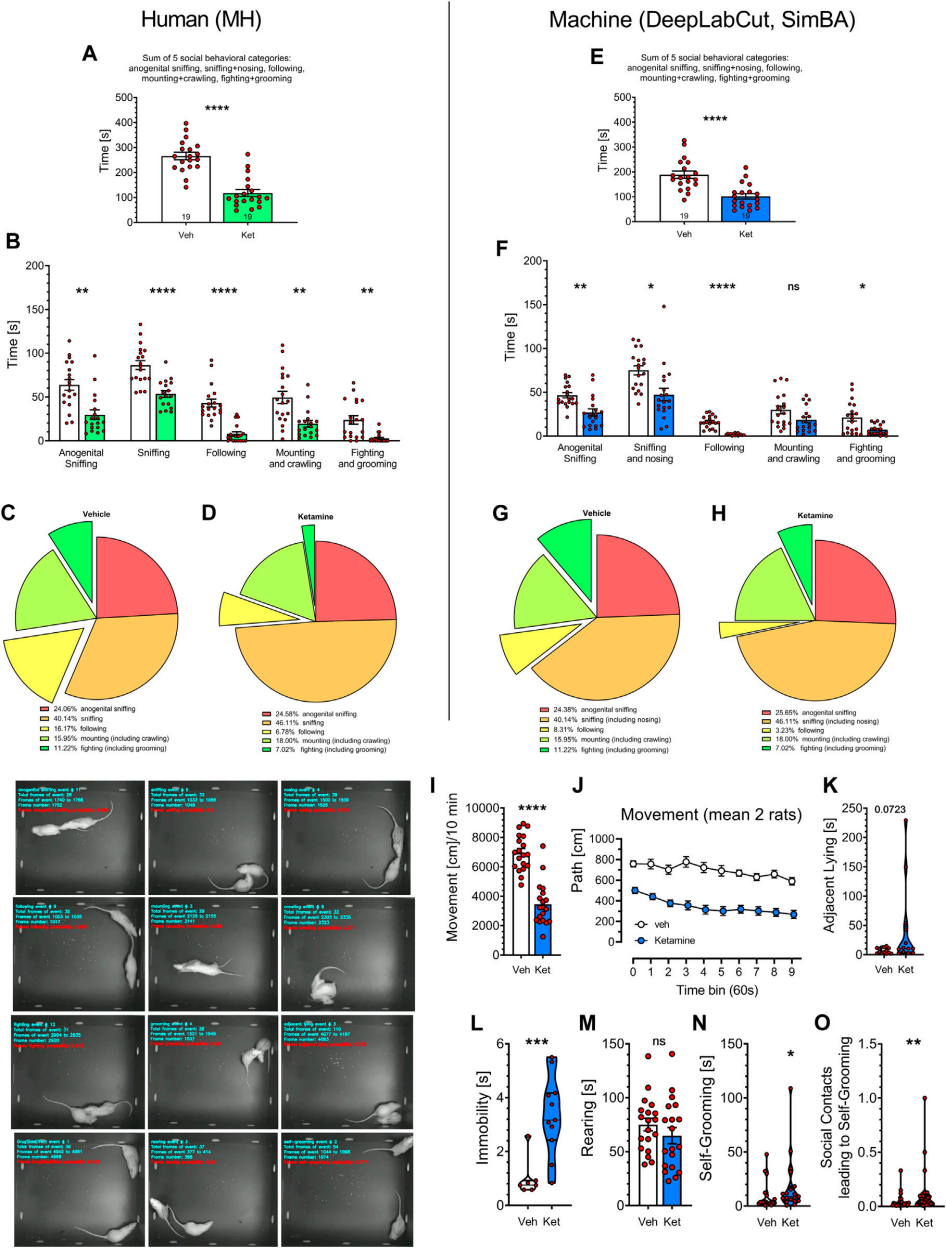
Statistical comparisons of historical videos with rat social behavior analyzed over the years 2017–2018 by MH (left panels) and by the digital workflow (DeepLabCut and SimBA toolkits; right panels). Nineteen videos of vehicle- and 19 videos of Vetoquinol ketamine-treated rats were included and their analyses were compared side-by-side. Panels **(A,E)** show the sum of time in seconds of social behaviors, typically presented in literature. Analyses of specific behavioral categories **(B,F)** demonstrate remarkable similarities between human and digital workflow’s as the *post hoc* analyses revealed decreases (*p* < 0.05–0.001) in all 7 behaviors analyzed in both datasets, except mounting + crawling, for which SimBA found no difference between Vetoquinol ketamine and vehicle groups. Treatment with ketamine appeared to reduce the percentage of fighting and following behaviors [**(C,D)** and **(G,H)**, respectively] as assessed by the human and digital workflows. Additional analyses with digital workflow only revealed a decrease of movement in rats treated with Vetoquinol ketamine **(I)**, being observed for the whole time of observation **(J)**, insignificant increase of adjacent lying **(K)**, significant increase of immobility **(L)** and self-grooming time **(N)** but no effect on rearing time **(M)**. Panel **(O)** shows increased coefficients of self-grooming following social contacts derived from FSTTC analysis. The images on the bottom left show 12 representative frames of behavioral categories analyzed by SimBA toolkit (an example of immobility being marked as Drug Side Effect). *Symbols*: Veh: vehicle, Ket: Vetoquinol ketamine. Asterisks and numerical values above bars indicate differences (P’s) toward vehicle conditions. Data distributed normally are shown as mean ± SEM; data lacking normal distribution are shown as the violin plots.

To compare the times of different behaviors detected by the human and SimBA classifiers, regression analyses (Figure 2) and statistical comparisons (Figures 3A–H) were calculated for given behavior. Because in the previous work we did not measure locomotion, adjacent lying, immobility, rearing and self-grooming, the direct comparison of historical and present datasets regarded behavioral categories that were present in historical assessments only. Nonetheless, the additional behavioral categories analyzed by SimBA were included in the statistical analyses (Figures 3I–N). Since SimBA allowed the analysis of FSTTC (Lee et al., 2019), we calculated how often the social contacts (anogenital sniffing, crawling, fighting, grooming, mounting, nosing, and sniffing) preceded self-grooming behavior in vehicle- and in Vetoquinol ketamine-treated rats (Figures 3O); see (Becker et al., 2014; Frantz et al., 2018).

#### 2.1.5 Analysis of novel data: Comparing the effects of Vetoquinol ketamine with (*R,S*)-ketamine on rat social behaviors

**FIGURE 4:**
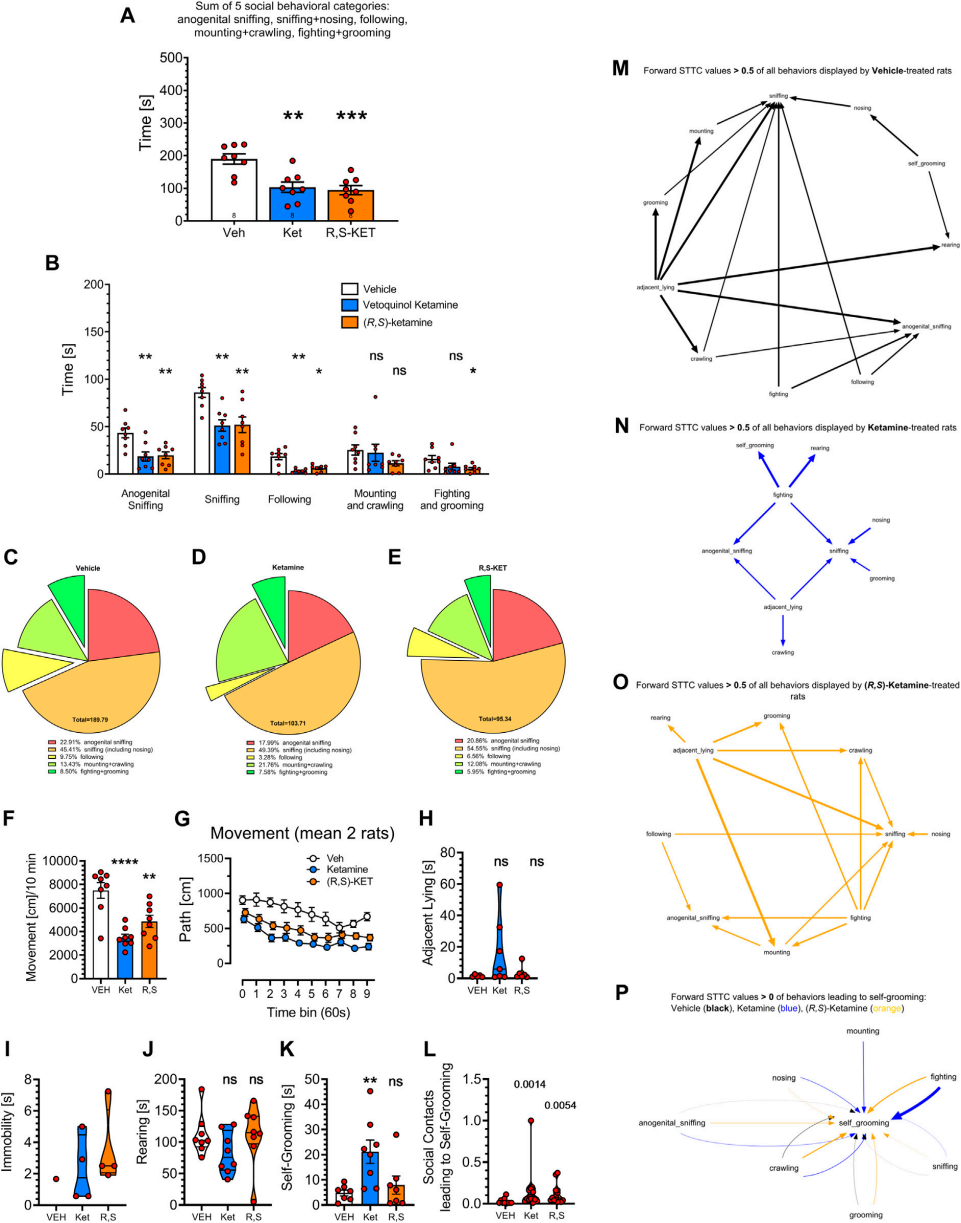
Effects of vehicle, Vetoquinol ketamine and pure (*R,S*)-ketamine (both at 20 mg/kg, 30 min before the test) on rat social behavior as analyzed by the digital workflow (DeepLabCut and SimBA toolkits). **(A)** shows the sum of time in seconds of social behaviors, typically presented in literature. Analyses of specific categories **(B)** demonstrate decreases of anogenital sniffing, body sniffing and following behaviors due to the treatment with both compounds, no effect on mounting + crawling and suppression of fighting + grooming induced by (*R,S*)-ketamine only. Treatment with ketamine preparations appeared to reduce the percentage of the following behavior due to Vetoquinol ketamine **(D)** and of fighting + grooming due to (*R,S*)-ketamine treatment **(E)** as compared to Vehicle **(C)**. Digital workflow revealed also a decrease of movement in rats treated with both ketamine preparations **(F)**, evident for the whole time of observation **(G)**, insignificant increase of adjacent lying **(H)**, and significant increase of self-grooming due to Vetoquinol ketamine **(K)**. There was no effect of either compound on the rearing time **(J)**, while the effect on immobility could not be calculated as only one control rat displayed this behavior **(I)**. Panel **(L)** shows that both ketamine preparations increased coefficients of self-grooming following social contacts derived from FSTTC analysis. **(M–O)** show the most prominent (FSTTC cut-off > 0.5) transitions of all behavioral categories and suggest that the most complex was behavior of vehicle treated rats, followed by behavior of (*R,S*)-ketamine treated animals, while Vetoquinol ketamine treated rats exhibited the least complex behavioral transitions. **(P)** shows all 7 social contact behaviors (anogenital sniffing, crawling, fighting, grooming, mounting, nosing, and sniffing) leading to self-grooming with FSTTC coefficients > 0. In Vetoquinol ketamine animals (blue) it was mostly fighting, mounting, nosing and crawling, while in (*R,S*)-ketamine animals (orange) it was fighting and crawling that led to self-grooming. *Symbols*: Veh: vehicle, Ket: Vetoquinol ketamine, (*R,S*)-KET: pure (*R,S*)-ketamine. Asterisks and numerical values above bars indicate differences (P’s) toward vehicle conditions. Data distributed normally are shown as mean ± SEM; data lacking normal distribution are shown as the violin plots.

Using most the features offered by SimBA, we analyzed similarities and differences between veterinary ketamine preparation Vetoquinol and the pure (*R,S*)-ketamine (Figure 4).

### 2.2 Animals

Twenty-four male 7-week-old Sprague Dawley rats (Charles River, Germany) weighing ∼ 250 g upon arrival were used. Animals were group housed (4 per cage) in the standard laboratory cages under standard colony A/C controlled conditions: room temperature 21°C ± 2°C, humidity (40%–50%), 12-h light/dark cycle (lights on: 06:00) with *ad libitum* access to water and lab chow.

### 2.3 Drugs

(*R,S*)-ketamine hydrochloride was purified from the Vetoquinol preparation by Dr. Ryszard Bugno at the Department of Medicinal Chemistry of the Maj Institute of Pharmacology as described earlier (Popik et al., 2022). (*R,S*)-ketamine was dissolved in sterile milliQ water prior to injection. Vetoquinol Biowet racemic ketamine hydrochloride (aqueous solution (115.34 mg/mL, Gorzów Wielkopolski, Poland) was diluted in distilled water to the appropriate concentration. The dose of ketamine preparations (20 mg/kg) was based on the earlier reports on rat social behavior from this laboratory (Nikiforuk et al., 2013; Holuj et al., 2015; Popik et al., 2017). Physiological saline was used as placebo. Drugs were administered intraperitoneally (IP) in the volume of 1 mL/kg, 30 min before the test. All solutions were made fresh at the day of testing.

### 2.4 Apparatus

The experiment was conducted in an open field arena (length x width x height: 67 × 57 × 30 cm) made of black Plexiglas. The arena was dimly illuminated with an indirect light of 18 Lux. The behavior of the rats was recorded using Axis network camera placed above the arena and connected to the Synology Surveillance station. The videos (mp4 H.264 encoded, 640 × 480 pixels, variable bitrate, image quality 3), were recorded at 12 frames per second (fps).

### 2.5 Procedure

The animals were handled and weighed, and the backsides of one-half of the animals were dyed with a gentian violet (2% Methylrosanilinium chloride) solution for compatibility with earlier studies. On the test day, to increase the level of social behavior, two unfamiliar rats of matched body weight (±5 g) were placed in the open field arena, and their behaviors were recorded for 10 min.

### 2.6 Experimental design

#### 2.6.1 Sample size

On designing the study, we performed sample size estimation (Perugini et al., 2018) using https://www.psychometrica.de/effect_size.html site and free to use G*Power (https://www.psychologie.hhu.de/arbeitsgruppen/allgemeine-psychologie-und-arbeitspsychologie/gpower) software. The primary outcome measure of the social behavior analysis is the total time of social interactions, and we calculated Cohen's d’s based on the results presented by Zajdel et al. (2018) on their Figure 23-SI. Thus, with pooled SD of 55.925 (2 groups, one treated with vehicle and another with ketamine), we obtained the Cohen's d’s of 3.672. Submission of the effect size d parameter of 3.672 into G*Power software with alpha of 0.05 and power (1-beta error probability) of 0.95 resulted with the requirement of a minimal total sample size of 8 pairs for 2 treatment groups. A similar analysis with the higher alpha of 0.001 yielded the requirement of a total number of 14 pairs. These numbers are consistent with the number of pairs used typically in our research (Nikiforuk et al., 2013; Holuj et al., 2015; Popik et al., 2017; Zajdel et al., 2018).

#### 2.6.2 Randomization and blinding

Eight rats pairs per treatment were tested between 09:00 and 14:00 with the treatments given in a random order, as follows: vehicle, (*R,S*)-KET, (*R,S*)-KET, Vetoquinol-KET (animals from housing cages A and B); (*R,S*)-KET, (*R,S*)-KET, Vetoquinol-KET, vehicle (animals from housing cages C and D); vehicle, (*R,S*)-KET, Vetoquinol-KET, vehicle (animals from housing cages E and F); Vetoquinol-KET, vehicle, vehicle, Vetoquinol-KET, (animals from housing cages G and H); (*R,S*)-KET, Vetoquinol-KET, vehicle, Vetoquinol-KET, (animals from housing cages I and J); (*R,S*)-KET, Vetoquinol-KET, (*R,S*)-KET, vehicle, (animals from housing cages K and L). Randomization was done using R language script and the “crossdes” package to randomly select treatment sequences and allocate rats among them. It served to avoid purported effects of the time of day on the treatment response. As shown, within a given housing cage, rats received different treatments, which were the same as the treatment of their conspecifics from another housing cage. All solutions were given blindly, by an experimenter unaware of the treatment conditions and not involved in data analyses.

### 2.7 Statistics

Historical and novel SimBA data (i.e., the times of social behavior) were first checked for normal distribution using Anderson-Darling, D'Agostino & Pearson, Shapiro-Wilk and Kolmogorov-Smirnov tests. Data distributed normally were analyzed with the Student’s t-test, one-way ANOVA followed by Tukey’s *post hoc* test or two-way ANOVA followed by Dunnet’s or Sidak’s *post hoc* tests and are shown as mean ± SEM. Data lacking normal distribution were analyzed with Mann-Whitney’s test or Kruskal-Wallis ANOVA (Cardinal and Aitken, 2006), followed by two-stage linear step-up procedure of Benjamini, Krieger and Yekutieli as the *post hoc* test, and are shown as the violin plots.

Simple linear regression was used to compare human and digital workflow. GraphPad Prism version 9.0 was used throughout. The alpha level was set at 0.05.

### 2.8 Quality system

At the time of performing this study, Department of Behavioral Neuroscience and Drug Development has been holding EQIPD (Bespalov et al., 2021); see (https://quality-preclinical-data.eu/) certificate no. PL-INS-DBNDD-11-2021-2 (Nov 12th, 2021 – Nov 30th, 2024); see https://paasp.net/new-eqipd-certified-research-unit/ and https://go-eqipd.org/about-eqipd/eqipd-certified-research-groups/. Thus, this study followed EQIPD guidelines: https://eqipd-toolbox.paasp.net/wiki/EQIPD_Quality_System for instance, it was carefully designed in advance with the estimation of the sample size, was blinded and randomized and was documented in detail at every stage of progress.

## 3 Results

### 3.1 Validation: comparing human annotations with SimBA classifiers in detecting rats social behaviors on historical videos

Figure 2 shows the results of regression analyses and remarkable similarities between human (MH) and SimBA classifications of 101 historical videos; these were from our unpublished experiments of animals treated with various compounds. *R* ^2^ values for anogenital sniffing, sniffing including nosing, following, mounting + crawling were: 0.66, 0.37, 0.61, and 0.43, respectively (all *p* values < 0.0001).

While fighting’s (*R* ^2^ = 0.22) and grooming’s (*R* ^2^ = 0.15) regression analyses were also significant (*p* < 0.001), it was apparent that the human classified fighting-like behavior less frequently than SimBA, and grooming-like behavior more frequently than SimBA (slopes: 0.055 and 1.31, respectively). Bearing similarities between these two behaviors (mostly conspecific’s fur chewing), likely difficult to distinguish for the human and SimBA, we decided to combine them into a fighting + grooming category, which yielded *R* ^2^ = 0.67, slope of 0.83 and *p* < 0.001. Of note, the regression of total time of social interactions yielded *R* ^2^ = 0.75, slope of 1.04 and *p* < 0.001 (N = 101), Figures 2H.

Figures 3A, E display the total time of social behaviors, presented as in the publications from this and other laboratories. The analysis of 19 Vetoquinol ketamine and 19 vehicle videos (unpublished subset from 101 historical videos) revealed marked decrease of the total time in ketamine group as determined by the human: t = 7.260, and SimBA: t = 4.749, respectively; both df = 36, *p* < 0.0001.

We further analyzed how specific behavioral categories affected the total time of social behavior. Two-way ANOVA demonstrated significant effects of behavior category: F (1.974, 57.25) = 46.53 and treatment: F (1, 59) = 74.02 (*p* < 0.001) but insignificant interaction: F (4, 116) = 0.7888 for human observations (Figures 3B). Similarly, significant effects of behavior category: F (2.148, 58.00) = 67.37 and treatment: F (1, 67) = 31.51 (*p* < 0.001) but insignificant interaction: F (4, 108) = 1.838 were found for SimBA classifications (Figures 3F). Small differences in the degrees of freedom are due to the Geisser-Greenhouse’s correction and to the fact that not all animals displayed all the behaviors, precluding the use of repeated measures design. Specific *post hoc* analyses revealed decreases (*p* < 0.05–0.001) in all 7 behaviors analyzed in both datasets, except mounting + crawling, for which SimBA found no difference between Vetoquinol ketamine and vehicle groups (Figures 3B, F). Treatment with ketamine appeared to reduce the percentage of fighting and following behaviors (Figures 3C, D, G, H, respectively) as assessed by the human and digital workflows.

SimBA’s-revealed decrease of movement in rats treated with Vetoquinol ketamine: t = 8.033, df = 36, *p* < 0.001 (Figure 3I) was noted for the whole time of observation: treatment factor F (1, 36) = 64.53; *p* < 0.0001; for every time point the Sidak’s *post hoc* test showed decrease of movement: *p* < 0.001 (Figure 3J).

The analyses of other SimBA’s measurements revealed that Vetoquinol ketamine insignificantly *increased* the time of adjacent lying: Mann-Whitney U = 49; *p* = 0.0723; (Figure 3K), significantly increased immobility: Mann-Whitney U = 4.500; p = 0.0008; (Figure 3L), did not affect rearing time (Figure 3M), but increased self-grooming time: Mann-Whitney U = 103; p = 0.0229 (Figure 3N) and the FSTTC coefficients of self-grooming following social contacts: Mann-Whitney U = 249; p = 0.0031 (Figure 3O).

Overall, both regression and direct analyses revealed almost identical reduction of social behaviors induced by Vetoquinol ketamine as assessed by the human (MH) and by the digital workflow.

### 3.2 Analysis of novel data: comparing the effects of Vetoquinol ketamine with (*R,S*)-ketamine on rat social behaviors

Having well-trained and validated classifiers, the novel 24 videos of rats treated with Vetoquinol and (*R,S*)-ketamine were analyzed using digital workflow only.

As shown on Figure 4A, treatment affected the total time of social behavior: F (2, 21) = 11.89; *p* = 0.0004 and the Tukey’s *post hoc* test revealed decreases due to the treatment with both compounds (*p* < 0.01–0.001) but no difference between them.

The analysis of specific behavioral categories affecting total time of social behavior revealed significant effects of the category: F (2.337, 47.90) = 80.39; *p* < 0.0001, treatment: F (2, 21) = 11.71; *p* = 0.0004 and an interaction: F (8, 82) = 2.689; *p* = 0.0112. Specific *post hoc* analyses with the Dunnett’s test revealed decreases (*p* < 0.01) in anogenital sniffing, sniffing, and following due to both ketamine preparations, no effects on mounting + crawling produced by both compounds, and a decrease of fighting + grooming due to (*R,S*)-ketamine; *p* < 0.05; Figure 4B. The lack of effect on mounting + crawling resembles similar observation in historical videos (see Figures 3B, F), alike reduced percentage of fighting and following behaviors (Figures 4C–E) due to the treatment with both compounds.

One-way ANOVA revealed that the treatments affected movement: F (2, 21) = 15.36; *p* < 0.0001 and the Tukey’s *post hoc* test showed reductions due to the treatment with Vetoquinol (*p* < 0.0001) and with (*R,S*)-ketamine (*p* = 0.0048), but no differences between ketamine preparations (Figure 4F). Movement reductions were noted for the whole time of observation: treatment factor: F (2, 21) = 14.99; *p* < 0.0001, mostly for Vetoquinol ketamine, at almost every time point (Figure 4G); the detailed results are not shown for the sake of figure clarity.

As with historical videos, Vetoquinol ketamine insignificantly increased adjacent lying (Figure 4H) and did not affect rearing (Figure 4J). In the present dataset it was not possible to assess the effects of ketamine preparations on immobility because only one of vehicle treated rats displayed that behavior (Figure 4I). The treatment affected self-grooming: F (2, 19) = 6.006; *p* = 0.0095 and the Dunnett’s *post hoc* test revealed that Vetoquinol ketamine but not (*R,S*)-ketamine increased this measure: *p* = 0.0082; Figure 4K. We also found that the treatments affected the FSTTC coefficients of self-grooming following social contacts: Kruskal-Wallis test = 11.47; *p* = 0.0032. Both Vetoquinol (*p* = 0.0014) and (*R,S*)-ketamine (*p* = 0.0054) increased this measure as assessed with two-stage linear step-up procedure of Benjamini, Krieger and Yekutieli (Figure 4L). Finally we attempted to visualize the complexity of all behavioral transitions, and Figures 4M–O shows that the most complex was behavior of vehicle treated rats, followed by behavior of (*R,S*)-ketamine treated animals, while Vetoquinol ketamine treated rats exhibited the least complex behavioral transitions. To elucidate the phenomenon of increased transitions leading to self-grooming (see Figure 4L), we plotted all 7 social contact behaviors (anogenital sniffing, crawling, fighting, grooming, mounting, nosing, and sniffing) leading to self-grooming, and noted that in Vetoquinol ketamine treated animals, it was mostly fighting, then mounting, nosing and crawling, while in (*R,S*)-ketamine animals it was fighting and crawling that led to self-grooming (Figure 4P).

Overall, (*R,S*)-ketamine was more efficient in reducing fighting + grooming and less efficient in increasing self-grooming than Vetoquinol ketamine, though there were no significant differences between the treatments. (*R,S*)-ketamine also resulted in an intermediate complexity of behavioral transitions. Other than that, both compounds displayed striking similarity in their effects on social behavior as we failed to find differences between their actions.

## 4 Discussion

Novel digital techniques including machine learning toolkits allow for rapid and objective measurements of animal behavior; for the reviews regarding DeepLabCut see (Hardin and Schlupp, 2022; Harrison, 2023). The SimBA open-source package with graphical interface and workflow has been used to characterize mouse and rat aggressive behavior (Nilsson et al., 2020) and more recently, rat maternal behavior (Lapp et al., 2023).

Present results demonstrate remarkable similarity between the experienced researcher’s annotations, and digital classifications in detecting rat social behaviors. Regression analyses of 101 videos showed that both workflows assigned almost the same time of social behaviors, with some differences in classifying the fighting and grooming behavior, likely due to the fact that they present similar features, that is, mostly conspecific’s fur licking and chewing. Except for not detecting ketamine-induced reduction of mounting + crawling categories by the digital workflow, all other possible effects of ketamine in 19 historical videos were nearly identical.

Of note, the DeepLabCut and SimBA toolkits are in our opinion easy to use even for the researcher not being fluent in Python’s packages implementations, do not require exceptional knowledge of deep learning/artificial intelligence algorithms and are supported by the helpful authors and Internet communities. In short, as of today, one does not have to be a computer “geek” to perform the analyses described here. On the other hand, present digital workflow required several months to develop working models of interacting animals, a fast computer with the fast graphics card, and numerous trial-and-error attempts to build satisfactory models.

The aim of the present work was to compare the effects of Vetoquinol ketamine preparation with the effects of pure (*R,S*)-ketamine on rat social behavior. This is because in our previous work (Nikiforuk et al., 2013; Holuj et al., 2015; Popik et al., 2017; Zajdel et al., 2018), we used a veterinary preparation of ketamine that contains also a minute (3 mg/mL) amount of the sedative compound chlorobutanol hemihydrate. Thus, a possibility existed that the preparation induced sedative effects, having little in common with the true pharmacological model of psychoses.

Present results, however, provide several grounds against the “sedation” hypothesis. First, the Vetoquinol preparation is typically used at the anesthetic doses (≥150 mg/kg) in animal surgery, and in the historic and novel studies we used it at the dose of 20 mg/kg, being ∼7 times lower. It could be hypothesized that the chlorobutanol’s concentration in the Vetoquinol ketamine was too low to induce sedation. Second, while the veterinary preparation reduced social behaviors, the antipsychotics amisulpride (3 mg/kg) (Nikiforuk et al., 2013); as well as aripiprazole (2 mg/kg) and risperidone (0.1 but not 0.3 mg/kg); (Zajdel et al., 2018), prevented for Vetoquinol ketamine - induced disruption of social behavior, increasing the total time of social behaviors. To our knowledge, these antipsychotic medications have no described stimulatory effect on rodent behavior. In addition, while Vetoquinol ketamine reduced locomotor activity, the pure (*R,S*)-ketamine displayed similar effects, and we found no direct differences between these ketamine preparations (Figures 4F, G). Lastly, the ketamine preparations did not affect another measure of the rat motion, i.e., the rearing behavior (Figure 4J). In short, the use of the “impure” Vetoquinol preparation appears as, as valid model of the “negative” symptoms of psychoses, as the pure (*R,S*)-ketamine.

While both human and digital workflows resulted in detecting similar total time of social behaviors in the controls (∼200–250 s per 10-min measurement), these values are ∼ two times lower than reported in our studies published in the years of 2013–2018 (Nikiforuk et al., 2013; Holuj et al., 2015; Popik et al., 2017; Zajdel et al., 2018). The reason of this discrepancy relies on the fact that, in previous experimental conditions, the animals were individually housed for 5 days prior to the start of the testing, to induce high level (∼400 s) of social interactions (Niesink and Van Ree, 1982). Because more recently we decided not to separate the animals, as the 5-day isolation could induce unnecessary separation stress, the total time of social interactions dropped to ∼ 200–300 s per 10-min measurement [see Figures 3A, B; Figure 4A and e.g., (Gzielo et al., 2020; Potasiewicz et al., 2020; Gzielo et al., 2023)].

How ketamine-induced social withdrawal corresponds with other models of human psychopathology? Present data showing ketamine-induced decrease of social behavior, at the first glance appear to provide little novel information. As with other NMDA and mGlu 5 receptor antagonists (Steinpreis et al., 1994; Sams-Dodd, 1995; Koros et al., 2007), ketamine preparations reduced time spent on social activities. However, thanks to the digital workflow, we examined here also behaviors not studied before, that is, adjacent lying and self-grooming. Adjacent lying (side by side contact or “huddling”) is regarded as a characteristic feature of the psychoactive (Nichols, 2016) MDMA “ecstasy” compound’s action (Morley et al., 2005; Ando et al., 2006). Analyzing both in the historical (Figure 3K) and novel videos (Figure 4H) we noted Vetoquinol-induced increases of this measure. Nonetheless, in both datasets the difference towards vehicle was not significant, suggesting that in the rat, ketamine does not produce MDMA-like actions. This is consistent with observations that ketamine produces hallucinogenic-like (Javitt and Zukin, 1991) but not MDMA-like actions (Nichols, 2016).

However, Vetoquinol ketamine increased the time of self-grooming (Figures 3N, 4K), regarded as highly stereotyped behavior (Kalueff et al., 2016), and both compounds (Figure 3O; Figures 4L, P) increased the transitions from the social contacts to self-grooming, as revealed by FSTTC analysis. This appears similar with the findings of other researchers (Becker et al., 2014; Frantz et al., 2018) showing that the time of self-grooming as well as the incidences of self-grooming following social contact were increased in mice lacking the mu opioid receptor (Oprm1^−/−^) that recapitulate the core symptoms of autism spectrum disorders (ASD). Other data from this laboratory demonstrate that in the rat neurodevelopmental model of ASD, based on maternal immune activation during pregnancy with polyinosinic:polycytidylic acid (poly (I:C)), the exposed offspring males, but not females spent more time on anogenital sniffing than the controls, suggestive of impaired social recognition (Gzielo et al., 2023). Since similar effect was not found in the present study, one can hypothesize that the ketamine preparations appeared not to resemble the poly (I:C) neurodevelopmental model of ASD. Ketamine produces social deficits also in mice (Faure et al., 2019). While several experimental details were different in this and the mouse study (lower dose of 10 mg/kg, social separation etc.,) the authors reported drug-induced decreases of affiliative behaviors (duration of social contact), dominance, following and escape behaviors, but intact rearing. Our findings (reduced fighting and following but intact rearing) appear to fit well with Faure et al. (2019) report.

Present digital workflow has some apparent limitations. The DeepLabCut and SimBA toolkits, in our hands did not allow to differentiate the interacting rats, thus, it was not possible to assign a given behavior to the A animal and the behavioral response to animal B. This caveat could be viewed particularly important in analyzing behavioral transitions. Second, in the human workflow one typically analyzes animal’s A behavior and then, separately animal’s B behavior, so the results are typically reported as the sum of the times of both animals’ behaviors. Present digital workflow assigned some categories like nosing, rearing and self-grooming as a single measure. This could lead to the assignment of less time spent in a given behavior than in the human workflow, when both animals were engaged in the same behavior. Finally, we are reporting the time but not the number of social behaviors. This was done on purpose, to compare published and present data, but also, to avoid increased numbers of analyses and figures, as these measures overlap (Gzielo et al., 2023).

In conclusion, we present a confirmatory study with an easy to replicate step-by-step workflow allowing digital measurement of rat social behavior, which, following validation on historical annotated videos, demonstrated similar actions of two ketamine preparations. In addition, we demonstrate that while ketamine has induced social withdrawal, it did not result in a MDMA-like effects, but that it may bear some similarities with the Oprm1^−/−^ mouse model of some symptoms of autism spectrum disorders.

## Data Availability

The original contributions presented in the study are included in the article/Supplementary Materials, further inquiries can be directed to the corresponding author. Additional files are available: http://gofile.me/5TcdS/S2bd8ei0p upon reasonable request (please contact the corresponding author).
